# Lumbar spine bone mineral density in women breastfeeding for a period of 4 to 6 months: systematic review and meta-analysis

**DOI:** 10.1186/s13006-023-00607-8

**Published:** 2023-12-18

**Authors:** Larissa Brazolotto Ferreira, Keny Gonçalves Tirapeli, Carla Cristiane Silva, Tamara Beres Lederer Goldberg

**Affiliations:** 1 Postgraduate Program in Tocogynecology, Botucatu Medical School, São Paulo State University (UNESP), Botucatu, São Paulo, Brazil; 2 Department of Human Movement Studies, Londrina State University (UEL), Londrina, Brazil

**Keywords:** Breastfeeding, Bone mineral density, Lactation

## Abstract

**Background:**

During the breastfeeding period, important transient changes in calcium homeostasis are verified in the maternal skeleton, to meet the demand for calcium for breastmilk production. The literature is inconclusive regarding the causes and percentages of involvement of bone densitometry resulting from exclusive breastfeeding (4 to 6 months).

**Methods:**

This article aims to systematically review the literature, to determine the occurrence, intensity, and factors involved in alterations in maternal bone mineral density (BMD), during a period of 4 to 6 months of exclusive breastfeeding. The search descriptors “*woman”*, “*breastfeeding*”, “*human milk*”, and “*bone mineral density*” were used in the electronic databases of the Virtual Health Library, Scielo (*Scientific Electronic Library Online*), CAPES Periodicals Portal, LILACS, Embase, PubMed/Medline, Cochrane, Scopus*,* and Web of Science in June 2023. Inclusion criteria for breastfeedingmothers were; aged to 40 years, primigravida, exclusively breastfeeding, with BMD assessments using dual-energy X-ray absorptiometry (DXA), with values expressed at baseline and from 4 to 6 months postpartum. The Jadad scale, Newcastle–Ottawa Scale, and Oxford Centre for Evidence-based Medicine – levels of evidence were adopted to assess the quality of the studies. For the meta-analytical study, statistical calculations were performed.

**Results:**

Initially, 381 articles were found using the search strategy and 26 were read in full. After risk of bias analysis, 16 articles remained in the systematic review and four were included in the meta-analysis. The studies showed a reduction in bone mass in the lumbar spine in the first months postpartum (4 – 6 months), when compared with a longer period of breastfeeding (12–18 months). The breastfeeding group presented a greater impact in the meta-analysis than the control group (non-breastfeeding, pregnant, or immediate postpartum), with a reduction in BMD in the lumbar spine of -0.18 g/cm^2^ (-0.36, -0.01 g/cm^2^); 95% Confidence Interval, on a scale from 0 to 10.

**Conclusions:**

Our results demonstrated a transitory reduction in bone densitometry of the lumbar spine during exclusive breastfeeding for 4 to 6 months, which was gradually restored later in the postpartum period. More prospective studies are needed to better understand the topic.

**Trial registration:**

PROSPERO platform (nº CRD42021279199), November 12th, 2021.

**Supplementary Information:**

The online version contains supplementary material available at 10.1186/s13006-023-00607-8.

## Background

During the breastfeeding period, important alterations occur in the homeostasis of calcium present in the maternal skeleton. These alterations are necessary to meet the high demand for this mineral directed to the production of breastmilk. Thus, through bone resorption, which is intensified in the maternal skeleton [1], around 200 to around 400 mg of calcium per day are removed from some breastfeeding mothers [1–5].

This mobilization is necessary to supply milk production according to the demands presented by the newborn and, subsequently, by the infant in the first months of life. If breastfeeding continues after six months, now added to solid foods, 120 mg of calcium/day will be needed from breast milk to meet the skeletal needs of the infant, and an additional 140 mg/day from food [6–8].

For the mother who exclusively breastfeeds, which is when the child receives only breastmilk, this process leads to a daily transfer of calcium through the milk, mobilized from the mother's skeleton, resulting from an increase in bone resorption, as well as, possibly, from an increase in intestinal absorption and a reduction in urinary excretion of the mineral during the breastfeeding period [1, 7, 9–12].

The transfer of calcium to breastmilk causes changes in bone mass. According to several studies, this transfer is evidenced by the decrease in bone mineral density (BMD) during the breastfeeding period, which can result in a reduction of up to 10% in BMD [13–15]. Although alterations in BMD are evident from the first postpartum months, the manifestation in different sites occurs with variable intensities and depends on the location analyzed [15]. It appears that the effects of breastfeeding on maternal bone mass are not homogeneous [12].

Several factors are involved in the way in which bone alterations appear in the postpartum period. These include changes in hormone levels, estrogen, parathyroid hormone (PTH) and prolactin, calcium and vitamin D consumption, number of births, maternal age, duration of breastfeeding, and ethnicity of mothers, among others [13].

Given these uncertainties and the multiple factors that can influence the outcome, when performing this type of analysis some variables severely impact the results presented. Studies that do not differentiate between the type of breastfeeding used (whether mixed, predominant, exclusive), the inclusion of multiparous and nulliparous women in the same group, postpartum follow-up for different times, and lack of differentiation when assessing BMD at the time when breastfeeding was suspended, interfere profoundly with the outcome.

Thus, the current systematic review with meta-analysis intends to report and standardize the results available in the literature, emphasizing that this is the first article to address the period from 4 to 6 months of exclusive breastfeeding, with the performance of a meta-analysis, proposing to determine the occurrence, intensity, and factors involved in alterations in BMD evidenced in primiparous women aged between 18 and 40 years.

## Methods

The objective is to indicate from this study “How much bone mass does the lactating woman lose during exclusive breastfeeding from 4 to 6 months postpartum?” This review was previously registered on the PROSPERO platform (nº CRD42021279199, November 12th, 2021). To this end, a search strategy was constructed to search for articles, through the prior definition of the Health Sciences Descriptors (DeCS) and database definitions. The descriptors used in the search, with the help of DeCS and of the Medical Subject Headings (MeSH), and of the standardized terms for Embase (Emtree) respectively, were: “*woman”*, “*breast feeding*”, “*milk human*”, “*bone mineral density*”. These were combined with the Boolean operators AND between keywords and OR between synonyms. Keywords were also used in the plural. The search strategy used is presented in the attachments (see Additional file 1).

The search strategy based on the descriptors and their synonyms was carried out in the electronic databases of the Virtual Health Library (VHL), Scielo (Scientific Electronic Library Online), CAPES Periodicals Portal, LILACS, Embase, PubMed/Medline, Cochrane, Scopus*,* and Web of Science. Database searches were performed using the VPN (Virtual Private Network) connected to UNESP in June 2023. This step was performed independently by the authors (LBF and KGT).

Subsequently, the results were arbitrated by a specialist, so that they could be compared and no articles would be missed. This capture step was also reviewed manually, with each title/abstract read by the authors. We chose to filter only articles in English, Portuguese, and Spanish; however, no filter was established for the period of time.

**Fig. 1:**
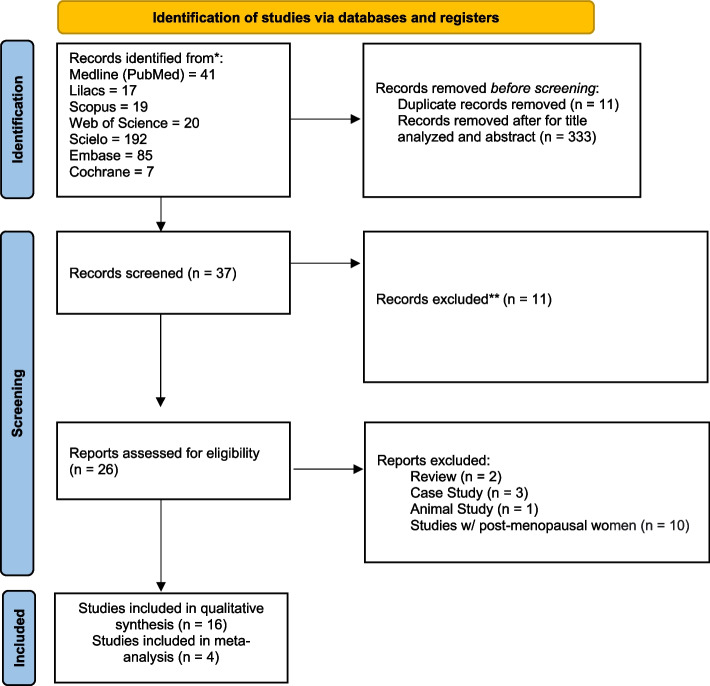
PRISMA diagram

To be included in this step, articles were required to contain at least one of the keywords or synonyms in the title and/or abstract. Filters present in the bases (text availability) were used, and in the sequence, the articles were selected and captured by two of the authors (LBF and KGT). Each researcher analyzed and read the whole group of articles (titles/abstracts). If there was disagreement, a third researcher (TBLG), who had also read the whole group of selected articles, evaluated and decided whether or not to include them. All the guidelines for Systematic Reviews were followed, such as the PRISMA Flow Diagram (Fig. 1) and the Cochrane Handbook for Systematic Reviews [16, 17].

### Eligibility and exclusion criteria

Regarding the selection of articles for this review, inclusion criteria were also determined for lactating mothers; aged between 18 and 40 years, thus including ages at which the Peak Bone Mass (PBM) had already been reached and the bone mass still remained at the plateau for women [18], primigravidas, exclusively breastfeeding, with a BMD assessment obtained by dual-energy X-ray absorptiometry (DXA), with values expressed at baseline and after 4 to 6 months postpartum. The breastfeeding mothers were required not to have been supplemented with calcium and/or to have engaged in strenuous physical activities, and not to be athletes, so that no other factors, other than the act of exclusively breastfeeding, could interfere with the assessment of their BMD.

Articles were selected based on titles, abstracts, and content, and were identified as eligible for inclusion if they met the following criteria: randomized controlled trials or observational studies (cohort, longitudinal, and cross-sectional), peer-reviewed studies, and studies reporting the occurrence, intensity, and factors involved in the changes in BMD evidenced in women during breastfeeding. Exclusion criteria were: articles without adjusted analysis; inadequate or undescribed sample selection; review articles; and duplicate articles, which had already been included in the search through another database.

This stage of the work was carried out by three independent researchers, blindly (LBF, KGT, TBLG). The complete articles were distributed to two researchers (LBF and KGT), and in case of disagreement, the inclusion or exclusion of the article was decided by the third researcher (TBLG). Subsequently, the selected articles were distributed again to the two researchers (KGT and LBF) for verification. The article references were analyzed in order to identify other publications of interest.

### Data collection

The information from the selected studies was extracted using a standardized Excel spreadsheet, to record all data and relevant variables. The data extracted were as follows: first author and year of publication, location, type of study, population, sample size, study and intervention groups, research duration, and main study results.

The level of scientific evidence and degree of recommendation of the included studies were determined according to the classification of the Oxford Center for Evidence-Based Medicine – Levels of evidence [19].

### Risk of bias analysis

The Jadad scale was adopted to assess the quality of the studies. This is a five-item tool used to report the risk of bias in clinical trials, through the assessment of; randomization, method of randomization, double blind, method of blinding, and reporting of losses and exclusions, respectively. The Jadad score ranges from 0 to 5, with values lower than 3 being considered low quality [20].

To assess the methodological quality of observational studies, the Newcastle–Ottawa Scale (NOS) was applied for cohort studies and the modified Newcastle–Ottawa Scale for cross-sectional and longitudinal studies [21–23]. The NOS is composed of 8 items. was used, in which each item can be given one star, except for the item "Comparability", where the score ranges from zero to two stars. The scale evaluates the studies based on criteria related to the selection and comparability between cohorts and criteria related to the study outcomes, with the objective of assessing the risk of bias based on the representativeness of the sample, exposure definition, presentation condition definition, response rate, and result determination. A low risk of bias is represented by a maximum score of nine stars for cohort studies and seven for cross-sectional and longitudinal studies. Cohort studies with six to eight stars were rated as moderate, and those with five stars or less were rated as low quality. Studies with good and moderate quality were included in the review. Cross-sectional and longitudinal studies with between four and six stars were rated as moderate, and those with three stars or less were considered as low quality [24].

The risk of bias assessments was carried out by the authors (LBF and KGT) and subsequently monitored. If there was any divergence in the scores, the evaluations were performed again, until full agreement was reached between the authors and there was no doubt about the evaluation.

### Meta-analysis

Numerical descriptive results are presented together with grouped data comparing BMD values between groups. The forest plot results are presented as the standardized mean difference (SMD) with the calculated 95% confidence interval, and the random effect was used to identify differences between groups. A meta-analysis on the primary data was only performed if two or more studies evaluated the same outcome of interest. For sensitivity analysis, studies with a higher risk of bias and, consequently, lower quality were removed one by one. The analysis allowed verification of the impact of combinations between the studies included in the meta-analysis and their explanatory power. The values for the effect of lactation were considered statistically significant when *P* < 0.05. Heterogeneity was also quantified with statistical I^2^, whereby 0–40% may not be important, 30–60% may represent moderate heterogeneity, 50–90% may represent high heterogeneity, and 75–100% is defined as considerable heterogeneity [17]. For the sensitivity analysis, studies with low or moderate methodological quality were excluded in accordance with the specific scales used related to the study design. Statistical calculations are illustrated by Forest plots constructed using the software *RevMan [Computer program]. Version 5.4.1 The Cochrane Collaboration* [17].

## Results

In total, 381 articles were found after applying the search strategy in the databases. Based on analysis of the titles and abstracts, 333 articles that were literature review articles, case studies, and studies carried out with experimental animals were excluded, in addition to 11 duplicate articles. Of the 37 remaining articles, 11 were excluded, for reasons described in Methods. Thus, 26 articles were analyzed by full reading. Of these, 16 articles were included in the systematic review, after risk of bias analysis, and were considered eligible for this review, as shown in the PRISMA diagram (Fig. 1) and four were included in the meta-analysis.

The studies included in this systematic review are presented in Table 1, with information on the authors, year, study location, population, and sample size. A supplementary document (see Additional file 2), contains complete data on the included studies, with the groups studied and results. Three studies were carried out in the USA [9, 25], three in the United Kingdom [1, 26, 27], one in Argentina [28], two in Mexico [29, 30], one in Denmark [12], one in Gambia [10], one in Israel [11], one in Sweden [31], one in China [5], one in Canada [32], and one in Thailand [33].

**Table 1:** Studies included in the systematic review

Author/year	Location	Population	Sample Size
Kalkwarf et al., 1997 [2]	Cincinnati, USA	Women (20 to 36 years old) with low or moderate calcium intake (≤ 800 mg/day), breastfeeding and offering artificial formula	326
Laskey et al., 1998 [1]	Cambridge, United Kingdom	Healthy white women (20 to 40 years old)	80
Ritchie et al., 1998 [9]	Berkeley, California, USA	Healthy women who consumed ≈1200 mg calcium/day (< 22 years or > 42 years)	14
Naylor et al., 2003 [26]	Sheffield, United Kingdom	Women who were planning to become pregnant (20 to 36 years old)	17
Glerean et al., 2010 [28]	Buenos Aires, Argentina	Primiparous and nulliparous women (21 to 40 years old)	61
Sámano et al., 2011 [29]	Mexico City, Mexico	Healthy women	72
Moller et al., 2012 [12]	Aarhus, Denmark	Healthy white women (25 to 35 years old)	228
Sawo et al., 2013 [10]	Keneba and Manduar villages in West Kiang countryside, The Gambia	Women (28.6 ± 8.4 years)	33
Lebel et al., 2014 [11]	Jerusalem, Israel	Women (20 to 46 years old)	132
Sámano et al., 2014 [30]	Mexico City, Mexico	women in the postpartum period	73
Brembeck et al., 2016 [31]	Gothenburg, Sweden	Pregnant women (25 to 40 years old)	81
Zhang et al., 2016 [5]	Guangzhou, China	Puerperal women (20 to 35 years old)	150
Cooke-Hubley et al., 2017 [32]	Newfoundland, Canada	Women who exclusively breastfed for 6 months	31
Teerapornpuntakit et al., 2017 [33]	Bangkok, Thailand	Healthy women (18 to 35 years old)	80
Cullers et al., 2019 [25]	Oakland and East Bay Area, California, USA	Women in the 16th week of pregnancy	64
Ó Breasail et al., 2020 [27]	Cambridge, United Kingdom	Healthy women (30 to 45 years old)	90

The majority of studies included in this systematic review used dual-energy X-ray absorptiometry (DXA) to obtain lumbar spine BMD, with two investigations [25, 27], in addition to DXA, the evaluation of BMD was performed by Peripheral Quantitative Computed Tomography (pQCT).

When analyzing the studies, we identified results obtained in several sites through the densitometric evaluation. Fourteen studies from the 16 articles included in this review, presented results for lumbar spine BMD, except the studies by Cullers et al. [25] and Ó Breasail et al. [27], which analyzed tibia and radius. Some studies included upper limb BMD (UL) described as radius, forearm, or wrist [2, 9, 12, 25–27]. Except for the study carried out by Kalkwarf et al. [2], all others identified BMD of lower limbs (LL), however, with a variety of areas analyzed, such as femur, hip, leg, tibia, trochanter, and even Ward's triangle. Some investigations evaluated total body BMD [1, 5, 9, 10, 12, 26].

Two studies compared the BMD of pregnant women with that of non-pregnant women [12, 27]; while Lebel et al. [11] evaluated nulliparous and multiparous women. Great variability was observed between the studies regarding the period of breastfeeding in which the densitometric analyses were performed. Analysis of breastfeeding and non-breastfeeding women was observed [1, 2]. Three studies evaluated pregnancy and different breastfeeding times: Ritchie et al. [9] (gestation, 6–10 weeks of breastfeeding, and after duration of postpartum amenorrhea 8 ± 3 months, at the resumption of menses (5 ± 2 months postmenses); Naylor et al. [26] (gestation, 15 days, and 3 months of breastfeeding); and Brembeck et al. [31], (gestation, 4, 12, and 18 months of breastfeeding). The remainder of the studies focused on the postpartum period of breastfeeding; Glerean et al. [28] chose to evaluate the immediate postpartum period, and 6 and 12 months of breastfeeding, Sámano et al. [29] and Sámano et al. [30] evaluated 15 days, and 3 and 6 months of breastfeeding; Cooke-Hubley et al. [32] evaluated 6 months and 12 months; and Teerapornpuntakit et al. [34] 6 months of breastfeeding.

In view of the diversity of study designs, the systematic review was complemented by a meta-analysis including a smaller number of studies. To improve effectiveness, studies were used that matched the basal periods (pregnancy and postpartum) with the period of exclusive breastfeeding (4 to 6 months), to answer the initial question: “Does exclusive breastfeeding during a period of 4 to 6 months change BMD?”.

However, despite having the objective of evaluating the differences in BMD in the various sites that can be analyzed, the literature only provides this information for the qualitative analysis, with greater focus on the DXA obtained in the lumbar spine. For the quantitative study (meta-analysis) only the lumbar spine was included, for the reasons already presented.

The results referring to the risk of bias analysis were divided according to the design of each of the studies: Table 2 describes the risk of bias assessment of randomized studies, Table 3 refers to the risk of bias assessment of cross-sectional and longitudinal studies, and Table 4 presents the risk of bias assessment of cohort studies (see Additional file 3 for supplementary Tables 2, 3, and 4). In Table 2, three randomized studies are identified Kalkwarf et al. [2]; Zhang et al. [5], and Cullers et al. [25]. The studies by Zhang et al. [5] and Cullers et al. [25] despite being included in the construction of the Systematic Review were not included in the meta-analysis, since Zhang et al. [5] analyze the BMD outside the period determined for this systematic review and meta-analysis and Cullers et al. [25] used pQCT to determine BMD. Regarding the risk of bias of cross-sectional and longitudinal studies (Table 3), only one study presented a low risk of bias, with a score equal to 7 [27], 5 studies showed a moderate risk, with a score of between 4 and 6 [10, 26, 28, 31, 33], and 2 studies [1, 9] showed low quality, however, some of the studies did not meet the other inclusion criteria that were described in the methods, which prevented their inclusion in the meta-analysis.

For the cohort studies (Table 4) it is noted that the studies by Moller et al. [12] and Cooke-Hubley et al. [32] presented a low risk of bias, and could be selected for the meta-analysis if they met the other proposed criteria. Only one of the cohort studies selected for this review scored 5, with low quality [11], so was not included in the meta-analysis. Considering the studies that presented moderate risk, with a score of 6 to 8 [29, 30], one was not included in the meta-analysis, since its results regarding densitometry were similar to those published at an earlier date [30].

Considering the information presented above, it was judged that a quantitative result could be obtained by carrying out a meta-analysis, with studies that contained BMD of the lumbar spine, since the studies by Kalkwarf et al. [2], Moller et al. [12], Sámano et al. [29], and Glerean et al. [28], presented the results at baseline and after 4 to 6 months of exclusive breastfeeding, as proposed in the inclusion criteria. Of the 16 studies that were selected for this review, 12 of them, despite being extremely informative, and contributing to the topic of breastfeeding versus bone mineral density, were not included in the meta-analysis, as they did not meet the criteria to demonstrate them as robust in terms of risk of bias or meeting the inclusion criteria.

Thus, only the studies by Glerean et al. [28]; Kalkwarf et al. [2]; Moller et al. [12], and Sámano et al. [29] met the quantitative criteria for the meta-analytic study in the primary analyses. The study by Cooke-Hubley et al., despite presenting a low risk of bias, was excluded from the meta-analysis because it did not provide baseline values [32]. The authors were contacted, but they did not supply these data.

**Fig. 2:**
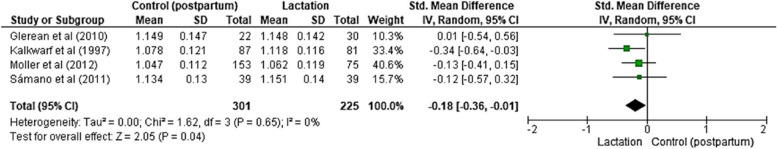
Forest plot of the meta-analyses of the effect of breastfeeding on lumbar spine. Note. Software RevMan [Computer program]. Version 5.4.1 The Cochrane Collaboration, 2020

Figure 2 indicates the forest plot of the meta-analytical study based on the results of the mean of the standardized differences and 95% confidence interval. BMD values in the lumbar spine (g/cm^2^) were plotted at the postpartum moment characterizing the control group and from 4 to 6 months of lactation, characterizing the lactation group**.**

**Fig. 3:**
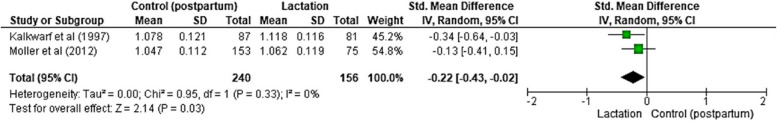
Sensitivity analyses of impact of the effects of breastfeeding excluding trials with more bias. Note. Software RevMan [Computer program]. Version 5.4.1 The Cochrane Collaboration, 2020

The results of the meta-analysis showed that the breastfeeding group presented a greater reduction in BMD than the control group (non-breastfeeding, pregnant, or immediate postpartum), with a reduction in BMD in the lumbar spine of [-0.18 g/cm^2^ (-0,36, -0,01 g/cm^2^); *P* = 0.04]. The greatest contribution to the results presented in this meta-analysis was the study by Moller et al. [12], with 40.6% weight followed by the study by Kalkwarf et al. with 33% [2]. Subsequently, a sensitivity analysis of the meta-analytic study was carried out, which consists of verifying whether, after removing studies with a higher risk of bias, the results are similar in direction, magnitude of effect, and statistical significance, which indicates a result robust meta-analysis. Thus, Fig. 3 shows the studies after sensitivity analysis, where the investigations by Glerean et al. [28] and Sámano et al. [29] were removed because they presented scores of 5 and 7 in the risk of bias analysis by the Newcastle–Ottawa Scale for cross-sectional and longitudinal studies, Tables 3 and 4 respectively (see Additional file 3).

The results of the sensitivity analysis, reinforced the reduction in BMD in the lumbar spine [-0.22 g/cm (-0,43; -0,02 g/cm^2^); *P* = 0.03], that is, the results of the meta-analysis (Fig. 2) are robust after passing through the sensitivity screening (Fig. 3). The larger effect size reflected the low risk of bias and, consequently, the quality of the studies involved, Kalkwarf et al. [2] and Moller et al. [12], which already indicated the highest weights in the primary analysis of the meta-analysis (Fig. 3).

Although the meta-analytical study included few studies that were eligible (*n* = 4) both in the primary analysis (*n* = 4) and in the sensitivity analysis (*n* = 2), no heterogeneity was observed (I^2^ = 0%). This value represents the percentage of variance attributed to heterogeneity, which is low or might not be important (0% to 40%) according to the Cochrane Collaboration Handbook [17]. In addition, it was observed that the studies in the meta-analysis did not demonstrate wide variation in confidence intervals.

## Discussion

This systematic review aimed to identify the changes that could occur in the BMD of lactating women who exclusively breastfed their children for a period of four to six months. However, it was observed that the majority of the analyzed studies presented results related to BMD of the lumbar spine, with a more restricted number of those that evaluated other skeletal sites, such as the total proximal femur, femoral neck, trochanteric region, hip, and whole body. The meta-analysis indicated that the breastfeeding group presented a greater impact in the outcomes than the control group (non-breastfeeding, pregnant, or immediate postpartum) with a lower standardized mean difference of BMD in the lumbar spine of -0.18 g/cm^2^ (-0.36, -0.01 g/cm^2^), *P* = 0.04. After sensitivity analysis, two high quality investigations remained in the meta-analytical study and reinforced the impact of the reduction in BMD in the lumbar spine [-0.22 g/cm^2^ (-0,43; -0,02 g/cm^2^), *P* = 0.03]. In addition to these points highlighted, not all the selected studies analyzed their results prospectively, with densitometry obtained at a minimum of two moments, and some studies included nursing mothers with different ages and nutritional which would compromise the analysis regarding the status, as well as, submitted to several factors that could influence the presented results, during the breastfeeding follow-up period, detection of any alteration in BMD in the investigated period, however, alterations could be detected in the lumbar region both by the systematic review and by the meta-analysis.

In general, the results indicated that involvement to the BMD occurred predominantly in the first postpartum months, during the period of exclusive breastfeeding, up to an average of four to six months. Pearson et al. observed a significant reduction in BMD in the spine, hip, and trochanter, with a more expressive reduction in the lumbar region. One year after delivery, most participants incorporated 5% of the preconception value, however, the recovery in the hip region was not similar [34]. Kovacs [7] pointed out that women who breastfed their children exclusively for 6 months presented a reduction in lumbar bone density of between 5 and 10% and a smaller percentage, perhaps half of that described, when sites richer in cortical bones were evaluated, demonstrating that the reduction did not occur homogeneously [7]. Considering the studies included in the current review [1, 2, 33] the results of lumbar spine BMD were lower than those evaluated in control women, who never breastfed. However, this reduction was observed at different times during the six months of breastfeeding, being more detectable in the lumbar region after between 4 and 6 months. Sámano et al. [29] identified that the BMD of the lumbar spine of women who breastfed up to 90 days postpartum presented lower results when compared to those obtained at 15 days postpartum. Teerapornpuntakit et al. [33] found a significant reduction in lumbar spine BMD among those who breastfed for three to four months, as well as persistence of the reduction in those who breastfed for up to six months. Thus, it is evident that the continuation of breastfeeding resulted in a negative impact on bone mass, which was related to the duration of exclusive breastfeeding and the analyzed site [31].

A study that evaluated BMD in the immediate postpartum period highlighted that lumbar spine BMD during this period was lower (5.2%) than in nulliparous controls, but without significant differences. Twelve months after delivery, the authors detected a statistically significant increase in relation to the evaluations obtained in the immediate postpartum period and after six months of breastfeeding. It is noteworthy that the BMD results at six months were practically stable in the lumbar, femur, and trochanter regions, when compared to the immediate postpartum period and close to those of the control participants [28]. If breastfeeding continues beyond the first semester, estradiol levels, which were previously low, tend to return to normal concentrations. In addition, the menstrual cycle resumes. The beginning of bone recovery in the nursing mother is associated with these factors, but also with the decrease in the volume of milk ingested by the infant due to the introduction of solid foods, and thus, less reabsorption of maternal bone mass occurs as a result of less calcium transfer for the child's skeleton [7]. There is loss of bone mass in the lumbar spine during pregnancy, in the postpartum period, and in the breastfeeding period, after which, approximately six months after delivery, there is stability and, from nine to 12 and up to 18 months, recovery of bone loss is reported, connoting the efficiency of the woman's hormonal negative feedback system [31]. Furthermore, it should be noted that parathyroid hormone-related protein (PTHrP) is found in very high concentrations in the blood of breastfeeding women and is produced in breast tissue, which results in increased maternal bone resorption and calcium absorption in the renal tubules and reduced excreted turnover. Calcium transported by breast milk is directed towards calcification of the skeleton of newborns and infants, which is the homeostatic mechanism suggested for compromising maternal bone mass in the first months of breastfeeding [7, 8].

Some of the selected studies identified the recovery of bone density loss in the lumbar spine over time, even if the lactating mother continued to breastfeed her child for a longer period of time. Among them, the study by Cooke-Hubley et al. stands out, which showed that the lumbar spine BMD increased by about 5%, when evaluated at 12 months postpartum, in relation to the BMD obtained among nursing mothers at six months postpartum. However, if the nursing mothers continued to breastfeed their children for a longer period than the six months proposed for weaning, even when supplemented with solid foods and infant formulas, the authors detected an increase in BMD, but to a lesser extent, in lumbar and thoracic spine. Unfortunately, in consultation with the authors, they did not obtain data in the immediate postpartum period, which prevented the inclusion of the study in our meta-analysis [32]. Another study compared women who were 18 months postpartum and identified loss of BMD in the lumbar spine in the fourth month of breastfeeding, with an increase in these values in the period of 12 and 18 months postpartum [31].

The study carried out by Moller and researchers [12] showed that the BMD was reduced during pregnancy compared to that of non-pregnant women. At 15 days postpartum, the authors observed a reduction of 2 to 3% in BMD in each of the analyzed sites, and in the lumbar spine this reduction increased to 5% in the continuity of exclusive breastfeeding for four months. When nursing mothers, still breastfeeding their children, non-exclusively, for a period longer than 9 months postpartum, performed a new DXA, they showed a 2% increase in lumbar spine BMD, and those who breastfed for a period of 4 to 9 months showed an increase of 4%, but the results were still lower than those obtained in the pre-gestational period. It took around 19 months postpartum for 2/3 of the densitometry of the monitored women, none of them breastfeeding their children, to return to pre-gestational values. These results reinforce that there is an association between the reduction in BMD according to the time and classification of breastfeeding, whether exclusive or complementary, and also, that if breastfeeding occurs for a long period (> 9 months), the recovery of BMD in the lumbar spine area seems to “progress” at a faster pace, while that detected in the whole body takes longer.

Regarding the possible preservation of the nursing mother's bone mass, there are also studies that investigated the effects of calcium and/or vitamin D supply in the diet, while some demonstrated positive effects on bone health, others do not corroborate these findings. Zhang and researchers [5] in a randomized double-blind controlled study with Chinese breastfeeding women, performed interventions, adding increasing doses of Ca for a period of 12 months, and analyzed the BMD of the total body, total left hip, and subregions, and lumbar spine (L1-L4). The authors concluded that there was no statistically significant difference in BMD and no beneficial effects of supplementation with increasing doses. In contrast, Cullers et al. [25] demonstrated that calcium supplementation during pregnancy influenced the bone recovery of women in the United States who ate a typical diet and that adequate calcium intake could prevent bone loss during lactation [35]. The recommendations for nursing mothers with reduced bone mineral density are within the parameters already described in the present review and adhere to the usually recommended doses of calcium by the Dietary Reference Intakes/ Recommended Dietary Allowance (DRIs/RDA), of 1000 mg/day for 19–50 years for pregnant/lactating women) [36] during the breastfeeding period, emphasizing that many women will gradually recover their bone density with the return of menstrual cycles and in a period of around 18 months postpartum [37].

The observed limitations resulted from the design of the studies and the inclusion criteria proposed for the articles consulted, since each study analyzed considered a different moment to be considered as baseline, with different follow-up times, which limited the inclusion of a greater number of studies in the meta-analysis. Another point to be highlighted is the lack of detailed information about obtaining BMD values, which interfered with the inclusion of a greater number of lactating mothers and controls in the meta-analysis. In addition, diversity of sample sizes was observed among the studies and many of them lacked a description of ethnicity/race and nutritional assessments of the nursing mothers included. Prospective studies are needed including a greater number of nursing mothers, and with control of factors that may interfere with the incorporation and resorption of bone mass. The various benefits of breastfeeding for the mother and child and also for the planet are unquestionable and well elucidated by the scientific community. However, it is known that breastfeeding rates around the world still need to be improved. There is a worldwide effort to extend the duration of this practice, according to global goals that involve the mobilization of public and private sectors.

Therefore, it is necessary to intensify public health actions that can promote bone mass improvement in women of reproductive age, especially during pregnancy and breastfeeding, such as adequate guidance on foods rich in calcium and vitamin D, timely exposure to sunlight, and the use of prophylactic nutrient supplementation.

## Conclusions

This systematic review followed by meta-analysis indicates that there is a transient reduction in bone mineral density in the lumbar region of women who breastfeed their children exclusively for 4 to 6 months. However, some prospective longitudinal studies suggest that bone density is gradually restored later in the postpartum period, without the need for weaning for this to occur. These results indicate the necessity to carry out more studies that improve understanding of the topic, due to the heterogeneity of the studies located, which made any quantitative compilation difficult.

### Supplementary Information

**Additional file 1: Appendix 1.** Search Strategies.

**Additional file 2:  Table 1. **a Complete findings of the selected articles included in the systematic review.

**Additional file 3: Supplementary Table 2.** Risk of Bias Assessment of Randomized Studies. **Supplementary Table 3.** Risk of Bias Assessment of Cross-sectional and Longitudinal Studies. ** Supplementary Table 4.** Risk of Bias Assessment of Cohort Studies.

## Abbreviations

aBMD: Areal bone mineral densitometry
BMD: Bone mineral density
DeCS: Health Sciences Descriptors
DRIs: Dietary Reference Intakes
DXA: Dual-energy X-ray absorptiometry
EMTREE: Standardized terms for Embase
FAPESP: Fundação de Amparo à Pesquisa do Estado de São Paulo
Fig.: Figure
HR-Pqct: High-resolution 3-dimensional peripheral quantitative computed tomography
LL: Lower limbs
MeSH: Medical Subject Headings
MRI: Magnetic resonance imaging
NOS: Newcastle-Ottawa Scale
OPG: Osteoprotegerin
PBM: Peak Bone Mass
pQCT: Peripheral Quantitative Computed Tomography
PTH: Parathyroid hormone
PTHrP: Parathyroid hormone-related protein
QUS: Quantitative ultrasound
RDA: Recommended Dietary Allowances
Scielo: Scientific Electronic Library Online
TNF: Tumor necrosis factor
UL: Upper limb
VHL: Virtual Health Library
VPN: Virtual Private Network
