# Supplementary material for: Lumbar spine bone mineral density in women breastfeeding for a period of 4 to 6 months: systematic review and meta-analysis
Source: Int Breastfeed J. 2023 Dec 18;18:68. doi: 10.1186/s13006-023-00607-8 (PMC10729562; doi:10.1186/s13006-023-00607-8)
Supplement: Supplementary file 1 — Additional file 1: Appendix 1. Search Strategies. [file 13006_2023_607_MOESM1_ESM.docx]

**APPENDIX 1 Search Strategies**

**DECS - Health Sciences Descriptors**

(Bone Density OR Densidad Ósea OR Densidade Óssea OR Conteúdo Mineral Ósseo OR Densidade Mineral Óssea OR Bone Densities OR Density, Bone OR Bone Mineral Density OR Bone Mineral Densities OR Density, Bone Mineral OR Bone Mineral Content OR Bone Mineral Contents) **AND** (Milk, Human OR Leche Humana OR Leite Humano OR Leite Materno OR Breast Milk OR Milk, Breast OR Human Milk) **AND** (Breast FeedingOR Feeding, Breast OR Breastfeeding OR Breast Feeding, Exclusive OR Exclusive Breast Feeding OR Breastfeeding, Exclusive OR Exclusive Breastfeeding OR Lactancia Materna OR Aleitamento Materno OR Aleitamento OR Alimentação ao Peito OR Amamentação OR Lactation OR Lactancia OR Lactação OR Lactação Prolongada OR Secreção de Leite OR Secreções de Leite OR Milk Secretion OR Milk Secretions OR Lactation, Prolonged OR Lactations, Prolonged OR Prolonged Lactation OR Prolonged Lactations)

**MESH - Medical Subject Headings**

("breast feeding" OR “breast feeding” OR “Feeding, Breast” OR Breastfeeding OR “Breast Feeding, Exclusive” OR “Exclusive Breast Feeding” OR “Breastfeeding, Exclusive” OR “Exclusive Breastfeeding” OR lactation OR lactations OR “Milk Secretion” OR “Milk Secretions” OR “Lactations, Prolonged” OR “Prolonged Lactation” OR “Prolonged Lactations”) **AND** ((((Milk Secretion OR Milk Secretions OR Lactation, Prolonged OR Lactations, Prolonged OR Prolonged Lactation OR Prolonged Lactations) OR "Lactation"[Mesh]) **AND** ("Bone Density" OR “Bone Densities” OR “Density, Bone” OR “Bone Mineral Density” OR “Bone Mineral Densities” OR “Density, Bone Mineral” OR “Bone Mineral Content” OR “Bone Mineral Contents” OR “Bone Marker” OR “Bone Markers”) **AND** ("women"[MeSH Terms] OR woman[Text Word])

**EMTREE - Standardized terms for Embase**

'breast feeding'/exp OR 'exclusive breastfeeding'/exp OR ''feeding, breast' OR 'breast feeding'/exp OR OR 'lactation'/exp OR 'breast secretion' OR 'lactic secretion' OR 'mammary gland secretion' OR 'milk excretion' OR 'milk release' OR 'milk secretion' OR 'lactation' **AND** 'bone density'/exp OR 'bone mineral density' OR 'density, bone' OR 'osseous density' OR 'bone density' OR 'bone mineral'/exp 'bone mineral content' OR 'bone mineral turnover' OR 'skeleton mineral' OR 'bone mineral' **AND** 'females' OR 'woman' OR 'women' OR 'female'
