# Supplementary material for: Lumbar spine bone mineral density in women breastfeeding for a period of 4 to 6 months: systematic review and meta-analysis
Source: Int Breastfeed J. 2023 Dec 18;18:68. doi: 10.1186/s13006-023-00607-8 (PMC10729562; doi:10.1186/s13006-023-00607-8)
Supplement: Supplementary file 3 — Additional file 3: Supplementary Table 2. Risk of Bias Assessment of Randomized Studies. Supplementary Table 3. Risk of Bias Assessment of Cross-sectional and Longitudinal Studies. Supplementary Table 4. Risk of Bias Assessment of Cohort Studies. [file 13006_2023_607_MOESM3_ESM.docx]

| **Table 2 Risk of Bias Assessment of Randomized Studies** | | | | | |
| --- | --- | --- | --- | --- | --- |
| **Author/year** | **Location** | **Type of Study** | **Jadad Scale Points^1^** | **Degree of recommendation^2^** | **Level of Evidence^2^** |
| **Kalkwarf et al., 1997** | Cincinnati, USA | Randomized  Double-blind  Placebo controlled  Prospective assessment between groups | 1 | A | 1B |
| **Zhang et al., 2016** | Guangzhou, China | Randomized  Double-blind  Controlled | 4 | A | 1B |
| **Cullers et al., 2019** | Oakland, California, USA | Randomized  Double-blind  Controlled | 1 | A | 1B |

^1^ Jadad AR, Moore RA, Carroll D, Jenkinson C, Reynolds DJ, Gavaghan DJ, McQuay HJ. Assessing the Quality of Reports of Randomized Clinical Trials: Is Blinding Necessary? Control Clin Trials. 1996 Feb;17(1):1-12.

^2^ OCEBM Levels of Evidence Working Group. Levels of Evidence (March 2009). Oxford Centre for Evidence-Based Medicine. Available at: <https://www.cebm.ox.ac.uk/resources/levels-of-evidence/oxford-centre-for-evidence-based-medicine-levels-of-evidence-march-2009>)

| **Table 3 Risk of Bias Assessment of Cross-sectional and Longitudinal Studies** | | | | | | | | |
| --- | --- | --- | --- | --- | --- | --- | --- | --- |
| **Author**  **/year** | **Type of Study** | **Selection** | | | **Comparability** | **Outcome** | |  |
|  |  | **Representativity** | **Response rate** | **Verification** |  | **Assessment** | **Statistical test** | **Total** |
| Teerapornpuntakit et al., 2017 | Cross-sectional | * | * |  | ** |  | * | 5 |
| Laskey et al., 1998 | Longitudinal | * | * |  |  |  | * | 3 |
| Ritchie et al., 1998 | Longitudinal | * | * |  |  |  | * | 3 |
| Naylor et al., 2003 | Longitudinal | * | * | * |  |  | * | 4 |
| Glerean et al., 2010 | Longitudinal | * | * |  | ** |  | * | 5 |
| Sawo et al., 2013 | Longitudinal | * | * | * |  |  | * | 4 |
| Brembeck et al., 2016 | Longitudinal | * | * | * | ** |  | * | 6 |
| Breasail et al., 2020 | Longitudinal | * | * | * | ** | * | * | 7 |

Newcastle-Ottawa Scale for cross-sectional and longitudinal studies.

| **Table 4 Risk of Bias Assessment of Cohort Studies** | | | | | | | | | |
| --- | --- | --- | --- | --- | --- | --- | --- | --- | --- |
| **Author**  **/year** | **SELECTION** | | | | **COMPARABILITY** | **OUTCOME** | | |  |
|  | **Representativity** | **Cohort selection not explained** | **Verification** | **End point not present at start** | **Based on design and analysis** | **Determination of the outcome** | **Length of follow-up** | **Follow-up adequacy** | **Total** |
| Sámano et al., 2011 | * | * | * | * |  | * | * | * | 7 |
| Moller et al., 2012 | * | * | * | * | ** | * | * | * | 9 |
| Lebel et al., 2014 | * | * | * | * |  | * |  |  | 5 |
| Sámano et al., 2014 | * | * | * | * |  | * | * | * | 7 |
| Cooke-Hubley, et al., 2017 | * | * | * | * | ** | * | * | * | 9 |

Newcastle-Ottawa Scale (NOS).
